# Prevalence and Factors Associated With Poor Sleep Quality Among Visitors of Primary Healthcare Centers in Al-Ahsa, Kingdom of Saudi Arabia: An Analytical Cross-Sectional Study

**DOI:** 10.7759/cureus.42653

**Published:** 2023-07-29

**Authors:** Abdullah A Albinsaleh, Walla M Al Wael, Manahil M Nouri, Ahmed M Alfayez, Mohammad H Alnasser, Mohammed J Alramadan

**Affiliations:** 1 Preventive Medicine, Al-Ahsa Health Cluster, Hofuf, SAU; 2 Physical Education, King Faisal University, Hofuf, SAU; 3 Community Medicine, University of Dongola, Dongola, SDN; 4 Medicine, Al-Ahsa Health Cluster, Hofuf, SAU; 5 Medicine, Ministry of Health, Hofuf, SAU

**Keywords:** mobile phone bedtime usage, primary healthcare centers, saudi arabia, al-ahsa, caffeine, smoking, anxiety, patient health questionnaire-2, depression, sleep quality

## Abstract

Introduction

Poor sleep quality can predict poor health and is associated with mortality risk. Many factors are associated with sleep quality such as gender, health, education, socioeconomic status, and stress. The objective of this study was to estimate the magnitude of poor sleep quality among visitors of Primary Healthcare Centers (PHCCs) in Al-Ahsa and to identify factors associated with poor sleep quality.

Methods

This is an analytical cross-sectional study. A multistage cluster sampling technique was used to recruit 461 visitors to PHCCs in Al-Ahsa Governorate in the Eastern Province of Saudi Arabia. A structured questionnaire was administered through face-to-face interviews. The questionnaire includes demographics, a validated Arabic version of the Pittsburgh Sleep Quality Index (PSQI), the Arabic version of the International Physical Activity Questionnaire (IPAQ), the Arabic version of the Patient Health Questionnaire-2 (PHQ-2), the Arabic version of the Generalized Anxiety Disorder-2 (GAD-2), the Arabic version of Perceived Stress Scale-10 (PSS-10), and a translated Mobile Related Sleep Risk Factors (MRSRF). Univariate analysis was performed using the Mann-Whitney U test for continuous data, the chi-square test (χ²) or Fishers's exact test (as appropriate) for categorical data, and logistic regression for multivariable analysis. A P-value of less than or equal to 0.05 was considered significant.

Results

The study included 433 participants, with 72.5% of them being poor sleepers (PSQI global score of over 5). The highest percentage of poor sleepers was found among those aged 18 - 28 years (81.7%), with no significant difference between genders (p = 0.676). The study's multivariable logistic regression analysis revealed that poor sleep is associated with smoking four hours before bedtime (OR = 2.9, CI = 1.2 - 6.7), consuming caffeine (drinks or pills) three hours before sleep (OR = 2.3, CI = 1.23 - 4.12) or immediately before bedtime (OR = 3.2, CI = 1.02 - 9.9), using mobile phones right before bedtime (OR = 2.6, CI = 1.5 - 4.5), having anxiety (OR = 5.8, CI = 1.3 - 26.2), and depression symptoms (OR = 6.5, CI = 2.9 - 14.5), among other risk factors.

Conclusion

The prevalence of poor sleep quality in our sample was notably high at 72.5%. Many factors are strongly associated with poor sleep quality including experiencing symptoms of anxiety and depression. Longitudinal studies are needed to explore this crucial health issue further. Healthcare providers in Al-Ahsa should pay particular attention while assessing patients who suffer from sleep disturbance by screening them for depression and anxiety and raising public awareness of the importance of good quality sleep and the factors that affect it.

## Introduction

Sleep is a vital biological process associated with human health and well-being. However, it is also related to personal behavior and social and environmental factors [1]. Sleep quality refers to how satisfied an individual is with their overall sleep experience. Sleep quality is a common term used in sleep medicine and covers various aspects of sleep. These aspects include metrics such as total sleep time, sleep maintenance, sleep efficiency, sleep onset latency, total wake time, and sometimes sleep disturbances like apnea or spontaneous arousal [2-3].

An individual's sleep quality could provide insight into their overall health, as there is an association between poor sleep and increased all-cause mortality and poor health [1]. Based on medical literature, the quality of sleep is associated with many factors such as gender, general health, educational background, socioeconomic status, and level of stress [4].

People of any age may experience stress, which can be defined as personal changes that lead to physical or psychological strain. The overall well-being of an individual will be based on his response to any factor that induces stress. Too much stress is an overwhelming experience that differs from individual to individual. Stress has many manifestations such as fear, inability to relax, and change in eating patterns, and can even lead to sleep disturbance [5]. In addition, psychiatric comorbidities such as depression and anxiety may impact sleep quality and daytime alertness, with a stronger impact on sleep when these mental conditions present together [6]. 

Smoking is among the factors that interfere with sleep quality, especially in individuals with high nicotine dependence. It is proven that sleep disturbance is likely to be more prevalent in smokers compared to non-smokers [7]. Due to the harmful effects of smoking, it was recommended to introduce sleep therapy as an adjunctive to smoking cessation programs [8].

Caffeine is one of the most widely popular psychoactive substances worldwide. People worldwide consume caffeine products, including drinks or tablets, to impede sleepiness and increase performance [9]. However, caffeine has adverse effects and attributes on sleep. Thus, it is advised to avoid caffeine consumption at night. In addition, caffeine consumption, while there is sleep insufficiency, may lead to impairment in initiating and maintaining sleep on the following days [10].

Another critical factor that is associated with sleep quality is physical activity. It is thought that exercise has a therapeutic behavioral side that aids sleep. Moreover, physical activity improves neuropsychological performance and sleep quality in older people [11]. Regardless of the intensity and mode of physical activity, it increases sleep efficiency and duration, especially in the middle-aged and elderly [12].

Technology has had a significant impact on human life. Many mobile users have smartphones which in turn causes poor sleep through many mechanisms. One is emitting blue light from the screens, which interferes with the circadian sleep rhythm and increases brain alertness, leading to poor sleep [13].

A night of balanced and good quality sleep is necessary for good quality of life. It is suggested through evidence that poor sleep leads to adverse medical and mental dysfunction. Many patients mistakenly attribute tiredness, daytime weakness, and loss of focus to family or social problems instead of the major cause, which is inadequate and unbalanced sleep [14].

Due to many factors, insufficient sleep is considered a public health epidemic that is not entirely recognized; it is underreported and has even a high economic burden. Several studies have measured poor sleep quality nationally and globally [15-21]. Studies conducted in Saudi Arabia have focused on specific groups with common medical conditions or small communities, such as university students [22]. None of the previous Saudi Studies have explored the association between poor sleep quality and stress and other factors in different age groups. Therefore, this study aims to assess the prevalence of poor sleep quality and the associated factors among visitors of Primary Healthcare Centers (PHCCs) in Al-Ahsa. Investigating such factors is crucial to create effective public health strategies that can alleviate its impact on health.

## Materials and methods

Study population and procedure

The study was conducted in Al-Ahsa Governorate, in the Eastern Province of Saudi Arabia. Al-Ahsa comprises four major cities: Al-Hofuf, Al-Mobarraz, Al-Oyoun, Al-Omran, and many small villages and hejar (remote villages). According to a 2021 estimate, Al-Ahsa has over 1,369,338 people among whom Saudi nationals constitute 80%. In Al-Ahsa Governorate, there are a total of 57 PHCCs that are distributed into four Health Sectors: The Northern, Southern, Central, and Eastern. PHCCs give free comprehensive health services to all Saudi nationals and specific groups of expatriates, including housemaids, drivers, and farmers. They provide curative (essential medications) and preventive measures (immunization, screening, antenatal care, health education, and public and environmental health services) to 550,816 registered people (provided by the healthcare information system). The study targeted visitors to these healthcare facilities. The Institutional Review Board of King Fahad Hospital, Hofuf (H-05-HS-065) issued approval - IRB Log No 85-EP-2022.

Inclusion criteria

In our study, because the included questionnaires had an age range of fifteen to 79 years, the participants must be at least fifteen years old.

Exclusion criteria

We excluded children whose parents did not accompany them during their visit to the PHCCs. Furthermore, pregnant women were not part of the study due to the intricate association between pregnancy and poor sleep quality, requiring an exclusive and more comprehensive investigation.

Sample size and sampling technique

The sample size was calculated using the following formula: n = N*X / (X + N - 1), where, X = Z_α/2_^2^*p*(1-p) / MOE^2^. Z_α/2_ is the critical value of the normal distribution at α/2 (the confidence (CI) level of 95%, α is 0.05, and 1.96 is the critical value). MOE is the margin of error (5%), p is the sample proportion (50%), and N is the PHCCs visitors population size (550,816). The minimum required sample size came to 385. In addition, we added 20% to compensate non-responders; the final sample size was 461 visitors to reach a representative sample of the primary healthcare population.

A multistage cluster sampling technique was used as follows: In the first stage, one PHCC from each of the four Al-Ahsa Health Sectors was randomly selected using the online application https://epitools.ausvet.com.au/randomnumbers. In the second stage, systematic random sampling was selected from the daily booked appointments and register list in each PHCC. The skip number between the visitors was generated electronically by Google dice roller.

Questionnaire

A paper-based, structured, Arabic-translated questionnaire was used during a face-to-face interview. The questionnaire comprised questions about sociodemographic characteristics, the patient's current smoking status, caffeine consumption, height, weight, chronic diseases, and family history of chronic diseases. In addition, we included previously validated Arabic version of the Pittsburgh Sleep Quality Index (PSQI), the Arabic version of the International Physical Activity Questionnaire (IPAQ), the Arabic version of the Patient Health Questionnaire-2 (PHQ-2), the Arabic version of Generalized Anxiety Disorder-2 (GAD-2), the Arabic version of Perceived Stress Scale-10 (PSS-10). Following the forward and backward translation technique, we translated Mobile Related Sleep Risk Factors (MRSRF) Questionnaire [13]. First, two English-Arabic professional translators independently translated it into Arabic. Second, four medical experts fluent in English-Arabic evaluated the translation. Third, two professional translators independently translated it into English. Finally, the panel evaluated the final translation and compared it to the original questionnaire. Two psychiatrists and two community health consultants performed content validation for the translated, and the other included questionnaires. We conducted a pilot study from January 5, 2023, to January 9, 2023, which involved 35 participants who completed all questionnaires. The reliability of these questionnaires ranged between 0.72 and 0.8 on the Cronbach alpha coefficient, indicating acceptable internal consistency.

Data collection and management

From January 22, 2023, to February 27, 2023, four well-trained personnel interviewed the PHCCs visitors, and the answers were collected and recorded systematically in electronic forms with automated export to Excel sheets and Statistical Package for Social Sciences (SPSS) (IBM Corp., Armonk, NY) software to ensure that the data was accurate and avoid missing ones.

Statistical analysis

Data management was performed using IBM SPSS, version 25. The Kolmogorov-Smirnov test was significant (p < 0.05), indicating that all the continuous data were not normally distributed. We analyzed the data using the Mann-Whitney U test for continuous data. In addition, the chi-square test (χ²) or Fisher's exact test was used as appropriate for categorical data. Data were summarized using mean and standard deviation (±S.D.) for numerical variables or frequency and percentage (%) for categorical variables. Then multivariate analyses were conducted using logistic regression to analyze the factors associated with poor sleep quality. A P-value of less than or equal to 0.05 was considered significant.

## Results

A total of 433 individuals agreed to participate (the response rate was 93%) and completed the questionnaire. Table 1 displays the characteristics of the participants, with 72.5% (n=314) being identified as poor sleepers (PSQI score above 5). The age group with the highest prevalence of poor sleep was 18-28 years, with 81.7% being identified as poor sleepers. Males comprised the majority of participants, accounting for 75% (n=325), with 72% (n=234) being identified as poor sleepers. However, there was no significant difference between males and females (p=0.676). There was a significant difference in educational level between the two groups (p=0.035). There was no statistical difference in the remaining sociodemographic characteristics of the participants, such as nationality, marital status, place of residence, job, family income, and transportation, between those in the poor sleep and good sleep groups.

**Table 1:** Demographic characteristics ^*^ Chi-square test. ^$ ^Differences between good sleepers and poor sleepers. ^**^ Fisher's exact test. ^+^ High school and pre-university diploma. ^&^ Middle school and pre-secondary school diploma.

Total (n= 433)			Pittsburgh Sleep Quality Index (PSQI) global score				
		n (%)	Good sleep quality (n = 119) 27.48%		Poor sleep quality (n = 314) 72.51%		P value^*$^
			n	%	n	%	
Age group	Less than 18	10 (2.3)	4	40%	6	60%	0.114
	18 - 28	82 (18.9)	15	18.3%	67	81.7%	
	29 - 39	155 (35.8)	38	24.5%	117	75.5%	
	40 - 50	138 (31.9)	44	31.9%	94	68.1%	
	51 - 59	31 (7.2)	11	35.5%	20	64.5%	
	60 and above	17 (3.9)	7	41.2%	10	58.8%	
Sex	Male	325 (75.1)	91	28%	234	72%	0.676
	Female	108 (24.9)	28	25.9%	80	74.1%	
Nationality	Saudi	426 (98.4)	116	27.2%	310	72.8%	0.399^**^
	Non-Saudi	7 (1.6)	3	42.9%	4	57.1%	
Marital Status	Married	334 (77.1)	98	29.3%	236	70.7%	0.112
	Widowed, Divorced, or Single	99 (22.9)	21	21.2%	78	78.8%	
Residence	Urban	321 (74.1)	87	27.1%	234	72.9%	0.764
	Rural	112 (25.9)	32	28.6%	80	71.4%	
Education	Postgraduate	62 (14.3)	18	29%	44	71%	0.035
	Bachelor's	143 (33)	35	24.5%	108	75.5%	
	High school^+^	173 (40)	46	26.6%	127	73.4%	
	Middle school^&^	34 (7.9)	8	23.5%	26	76.5%	
	Elementary school and less	21 (4.8)	12	57.1%	9	42.9%	
Job	Government Sector	135 (31.2)	41	30.4%	94	69.6%	0.981
	Private	126 (29.1)	34	27%	92	73%	
	Freelancer	25 (5.8)	7	28%	18	72%	
	Retired	24 (5.5)	6	25%	18	75%	
	Unemployed	23 (5.3)	6	26.1%	17	73.9%	
	Householder	67 (15.5)	16	23.9%	51	76.1%	
	Student	33 (7.6)	9	27.3%	24	72.7%	
Family Income (monthly)	Less than 15000 SR	331 (76.4)	87	26.3%	244	73.7%	0.314
	More than 15000 SR	102 (23.6)	32	31.4%	70	68.6%	
Transportation	Drives own vehicle	326 (75.3)	88	27%	238	73%	0.838
	With husband or family	87 (20.1)	26	29.9%	61	70.1%	
	Uber, Careem or by taxi	20 (4.6)	5	25%	15	75%	

Out of all the participants, 24.7% were smokers. Among this group, 81 individuals (75.7%) who smoked were poor sleepers. The smoking status, the number of cigarettes, or shishas were not statistically significant. The number of caffeine-containing drinks (coffee, tea, soft drinks, and energy drinks) and caffeine-containing pills had no statistical difference between individuals with poor-quality sleep and those with good-quality sleep. Among those who consume caffeine in the form of drinks or pills, the prevalence of poor-quality sleep increases with closer intake to bedtime (p = 0.009). The body mass index and physical level measured by IPAQ were not statistically significant between the two groups (Table 2). Regarding the number of hours spent on mobile phones watching YouTube videos or other programs, the total number of hours in a 24-hour period using a mobile phone screen, using the mobile phone right before bedtime, and using blue light filters on the mobile phone were all associated with poor sleep (p ≤ 0.003). The remaining answers regarding mobile phone usage were not statistically significant (Table 3).

**Table 2:** Lifestyle characteristics of the participants ^* ^Differences between good sleepers and poor sleepers.^#^ Mann-Whitney U tests. Else chi-square test.

Total (n= 433)			Pittsburgh Sleep Quality Index (PSQI) global score				
		n (%)	Good sleep quality (n= 119) 27.48%		Poor sleep quality (n= 314) 72.51%		P value^*^
			n/ Mean ±S.D.	%	n/ Mean ±S.D.	%	
Do you smoke?	Yes	107 (24.7)	26	24.3%	81	75.7%	0.123
	Ex-Smoker	29 (6.7)	4	13.8%	25	86.2%	
	No	297 (68.6)	89	30%	208	70%	
How many cigarettes per day?			2 ±6		3 ±7		0.307^#^
How many Shisha do you smoke per day?			0 ±1		0 ±1		0.160^#^
How many hours before bedtime do you smoke?	Four hours before sleep	53 (12.2)	8	15.1%	45	84.9%	0.096
	Immediately before sleep	60 (13.9)	17	28.3%	43	71.7%	
	I do not smoke	320 (73.9)	94	29.4%	226	70.6%	
How many cups of coffee do you drink per day?			1 ±2		1 ±2		0.537^#^
How many cups of tea do you drink per day?			3 ±2.3		2 ±2.9		0.273^#^
How many cans of soft drinks do you drink per day?			0.66 ±1.03		0.74 ±1.10		0.321^#^
How many cans of energy drinks do you drink per day?			0		0		0.765^#^
How many pills containing caffeine, do you take per day?			0		0 ±1		0.187^#^
How many hours before bedtime do you take any of the previous? (Caffeine-containing drinks and caffeine-containing pills)	More than 6 hours of sleep	30 (6.9)	10	33.3%	20	66.7%	0.009
	Before 6 hours of sleep	29 (6.7)	5	17.2%	24	82.8%	
	before 3 hours of sleep	111 (25.6)	23	20.7%	88	79.3%	
	Immediately before sleep	35 (8.1)	4	11.4%	31	88.6%	
	I do not drink or take.	228 (52.7)	77	33.8%	151	66.2%	
IPAQ	Inactive	338 (78.1)	91	26.9%	247	73.1%	0.623
	Active	95 (21.9)	28	29.5%	67	70.5%	
BMI score	Underweight	18 (4.2)	7	38.9%	11	61.1%	0.369
	Normal	152 (35.1)	41	27%	111	73%	
	Overweight	137 (31.6)	42	30.7%	95	69.3%	
	Obese	126 (29.1)	29	23%	97	77%	

**Table 3:** Mobile usage characteristics of the participants ^*^ Differences between good sleepers and poor sleepers. ^#^ Mann-Whitney U tests. Else chi-square test.

Total (n= 433)			Pittsburgh Sleep Quality Index (PSQI) global score				
		n (%)	Good sleep quality (n= 119) 27.48%		Poor sleep quality (n= 314) 72.51%		P value^*^
			n/ Mean ±S.D.	%	n/ Mean ±S.D.	%	
How many hours do you watch YouTube videos or other programs on your mobile phone?			6 ±4		7 ±4		0.003^#^
How many total hours in 24 hours do you use the mobile phone screen?			3.6 ±3.6		4.3 ±3.7		<0.001^#^
Do you use the mobile phone right before bed (while you are in bed when the lights are off?	Yes	347 (80.1)	79	22.8%	268	77.2%	<0.001
	No	86 (19.9)	40	46.5%	46	53.5%	
Do you keep your mobile phone on your bed (near your pillow) while you sleep?	Yes	321 (74.1)	82	25.5%	239	74.5%	0.126
	No	112 (25.9)	37	33%	75	67%	
Do you keep your mobile phone away from your bed (at least two meters away) while you sleep?	Yes	127 (29.3)	37	29.1%	90	70.9%	0.620
	No	306 (70.7)	82	26.8%	224	73.2%	
Do you put your mobile phone on silent while sleeping?	Yes	188 (43.3)	51	27.1%	137	72.9%	0.885
	No	245 (56.5)	68	27.8%	177	72.2%	
Do you put your mobile phone on airplane mode while you sleep?	Yes	64 (14.8)	12	18.8%	52	81.3%	0.090
	No	369 (85.2)	107	29%	262	71%	
Do you use your mobile phone's blue light filters (night mode)?	Yes	176 (40.6)	34	19.3%	142	80.7%	0.002
	No	257 (59.4)	85	33.1%	172	66.9%	

Among the total number of participants, 156 (36%) reported having one or more chronic diseases. These individuals were found to have a higher likelihood of experiencing poor sleep quality (p = 0.008). However, there was no significant difference in the prevalence of poor sleep quality in those with a family history of chronic diseases compared to those without any history. Moreover, those who tested positive for stress, depression, and anxiety through screening tools were found to have a higher prevalence of poor sleep quality (p <.001), as demonstrated in Table 4.

**Table 4:** Disease characteristics of the participants ^&^ Differences between good sleepers and poor sleepers. ^*^ Chi-square test. ^**^ Chronic diseases: anxiety was the only statistically significant variable by chi-square test (p <0.001) among a list of diseases. ^$ ^The participants were asked if they had any of the following: diabetes mellitus, hypertension, bronchial asthma, heart disease, neurological disease, endocrine disease, sickle cell disease, thalassemia, depression, anxiety, or cancer.

Total (n= 433)			Pittsburgh Sleep Quality Index (PSQI) global score				
		n (%)	Good sleep quality (n= 119) 27.48%		Poor sleep quality (n= 314) 72.51%		P value^&^*
			n	%	n	%	
Chronic Diseases^**$^	Yes	156 (36)	31	19.9%	125	80.1%	0.008
	No	277 (64)	88	31.8%	189	68.2%	
Family history of chronic diseases	Yes	378 (87.3)	103	27.2%	275	72.8%	0.775
	No	55 (12.7)	16	29.1%	39	70.9%	
PSS-10 score	Low stress (scores 0 - 13)	166 (38.3)	65	39.2%	101	60.8%	<0.001
	Moderate stress (scores 14 - 26)	243 (56.1)	51	21%	192	79%	
	High perceived stress (scores 27 - 40)	24 (5.5)	3	12.5%	21	87.5%	
PHQ-2 score	Normal	317 (73.2)	111	35%	206	65%	<0.001
	Depression (score of 3 or greater)	116 (26.8)	8	6.9%	108	93.1%	
GAD2 score	Normal	331 (76.4)	107	32.3%	224	67.7%	<0.001
	Anxiety (score of 3 or greater)	102 (23.6)	12	11.8%	90	88.2%	

Analysis of factors influencing sleep quality

In Table 5, we performed multivariable logistic regression with a backward stepwise drop of insignificant factors using the likelihood method to examine all the factors that showed a statistically significant association (P-value ≤ 0.10) in the univariate analysis. A preliminary analysis suggests that the assumption of multicollinearity was met (all variables had a tolerance of more than 0.1 and VIF was less than 5). The logistic model was statistically significant, χ² (10, N=433) = 92.2, p <0.001, which suggests that the model could distinguish between those with poor and good quality sleep. The model explained 27.7% (Nagelkerke R2) of the variance in the dependent variables and correctly classified 76.7% of the cases. Individuals who smoked a few hours before sleep were two times more likely to be poor sleepers (OR = 2.9, CI = 1.2 - 6.7). Regarding consuming caffeine-containing drinks and pills more than and before six hours of sleep, they had no statistically significant difference between both groups. However, those who took them before three hours were 2.3 times more likely to be poor sleepers (OR = 2.3, CI = 1.23 - 4.12). Furthermore, those who consumed the latter-mentioned substances immediately before sleep were 3.2 times more likely to be poor sleepers (OR = 3.2, CI = 1.02 - 9.9). The respondents who spent time on their mobile phones right before bedtime (while in bed when the lights were off) were 2.6 times more likely to have poor sleep quality (OR = 2.6, CI = 1.5 - 4.5). Moreover, those who used blue light filters (night mode) on their mobile phone were two times more likely to have poor sleep quality (OR = 2.1, CI = 1.3 - 3.5). Individuals who reported having anxiety were 5.8 times more likely to be poor sleepers (OR = 5.8, CI = 1.3 - 26.2). Participants with a positive score on the PHQ-2 test were 6.5 times more likely to have poor sleep quality (OR = 6.5, CI = 2.9 - 14.5). The remaining independent variables were not statistically significant.

**Table 5:** Multivariable logistic regression of factors influencing sleep quality among visitors of Primary Healthcare Centers (PHCCs) in Al-Ahsa

		P value	Odds ratio (OR)	95% C.I. for OR	
				Lower	Upper
How many hours before bedtime do you smoke? (Reference; I do not smoke)	Four hours before sleep	0.016	2.851	1.218	6.672
	Immediately before sleep	0.697	0.870	0.433	1.750
How many hours before bedtime do you take any of the previous? (Reference; I do not drink or take)	More than 6 hours of sleep	0.151	0.492	0.187	1.295
	Before 6 hours of sleep	0.173	2.107	0.722	6.152
	before 3 hours of sleep	0.008	2.253	1.232	4.120
	Immediately before sleep	0.046	3.174	1.019	9.883
Do you use the mobile phone right before bed (while you are in bed when the lights are off? (Reference; No)		0.001	2.597	1.492	4.518
Do you use your mobile phone's blue light filters (night mode)? (Reference; No)		0.003	2.139	1.293	3.538
Anxiety (Reference; No)		0.022	5.824	1.297	26.154
PHQ2 score (Reference; Normal)		<0.001	6.479	2.889	14.532

Analysis of sleep quality among visitors of PHCCs in Al-Ahsa

Table 6 (Appendices section) shows descriptive statistics of the PSQI global score and PSQI components. Among the 433 participants, the individuals who scored more than 5 in PSQI global score were 72.5% (n = 314). Only 29.8% (n = 129) reported very good in the subjective sleep quality component, and 13.4% (n = 58) had no difficulties in the sleep latency component. Fifty-one participants had < 5 hours of sleep, and 31 individuals had < 65% in the habitual sleep efficiency component. Twenty-one participants (4.8%) had no sleep disturbance, and most participants (79%) did not use sleep medications. In the daytime dysfunction, 97 individuals (22.4%) did their daily work and chores without difficulty. Table 7 (Appendices section) illustrates the PSQI components according to the poor and good sleep quality groups in depth.

## Discussion

In our study, the objectives were to estimate the magnitude of poor sleep quality among visitors of primary healthcare centers in Al-Ahsa and to identify factors associated with poor sleep among them. Based on our findings, the prevalence of poor sleep quality was 72.5% (n= 314) which is notably high compared to many studies addressing the same health issue. Two studies from Spain revealed a prevalence of poor sleep between 38.2% to about 50% [15-16]. Studies conducted in Korea showed poor sleep quality in 38% to 40% of the participants [17-18]. Ethiopia had a higher overall prevalence of poor sleep quality of 65.5% [19]. In Makkah and Riyadh, Saudi Arabia, the prevalence was 38.2% and 68%, respectively [20-21]. The differences in sampling technique, study area, and inclusion criteria could explain this variation in the prevalence across these studies.

Interestingly, those who smoked their last cigarette four hours before bedtime had a significant association with poor sleep quality, increasing the likelihood of poor sleep (OR = 2.9, CI = 1.2 - 6.7). Previous studies have shown that smoking, particularly a few hours before bedtime, is associated with poor sleep quality and fragmentation [23-24]. This association may be due to nicotine dependence, as individuals who wait a long time between smoking and going to bed tend to experience poor sleep quality.

We found that poor sleep quality was strongly associated with caffeine consumption time, which was statistically significant when taking them three hours (OR = 2.3, CI = 1.23 - 4.12) and immediately before bedtime (OR = 3.2, CI = 1.02 - 9.9). A study showed that consuming a moderate dose of caffeine at bedtime, three and six hours before bedtime, significantly affected sleep [25]. A systematic review and meta-analysis concluded that coffee should be consumed at least 8.8 hours before bedtime for an individual to avoid sleep disturbance [10].

In addition, our results showed that spending time on mobile phones right before bedtime had a statistically significant association with poor sleep quality (OR = 2.6, CI = 1.5 - 4.5). This finding goes along with another study that showed a negative impact on sleep between bedtime mobile phone usage and sleep quality [26]. Our study also shows that using blue light filters in mobiles increases the risk of poor-quality sleep (OR = 2.1, CI = 1.3 - 3.5). A possible explanation might be that people with sleep problems tend to use blue light filters to overcome them.

Individuals who reported having anxiety were 5.8 times more likely to be poor sleepers (OR = 5.8, CI = 1.3- 26.2) than those without anxiety. A study in Germany concluded that there is a moderate relationship between anxiety and sleep quality [27]. A positive screening result of depression by PHQ-2 had a statistically significant association with poor sleep quality (OR = 6.5, CI = 2.9 - 14.5). This finding supports that individuals with major depressive disorders tend to have sleep quality issues [28]. Because of the type of our study, temporality is challenging to conclude; a Korean study found that poor sleep quality may contribute to depressive symptoms, and screening and implementing measures to improve sleep may reduce the odds of having depression, thus; longitudinal studies are needed to prove which contributes to the other [29].

Overall, the results of our study will help aid preventive medicine, family medicine physicians, and other related specialties in assessing their patients who suffer from sleep disturbance by screening them for depression and anxiety and advising them to change their lifestyle habits of smoking and consuming caffeine-containing drinks and pills close to bedtime. In addition, public health and community medicine professionals should advocate for the dangers of mobile phone usage, especially its burden on sleep quality.

This study has a few limitations. Despite examining many factors and variables that may influence sleep quality, other important variables were not assessed including taking naps during the day, sleep environment, and night shift work. In addition, the cross-sectional nature of this study is a limitation, as causation cannot be concluded. Nevertheless, this study clearly shows the magnitude of poor-quality sleep and its association with a number of important factors.

## Conclusions

The prevalence of poor sleep quality in our sample was notably high at 72.5%. Many factors are strongly associated with poor sleep quality, including experiencing symptoms of anxiety and depression. Longitudinal studies are needed to explore this crucial health issue further. Physicians should pay particular attention while assessing patients who suffer from sleep disturbance by screening them for depression and anxiety and advising them to hold consuming caffeine until bedtime. In addition, advocacy for the dangers of mobile phone usage, especially its burden on sleep quality, should be met by public health and community medicine professionals.

## Appendices

**Table 6:** Descriptive statistics of PSQI global score and PSQI components

			n	%
PSQI global score		Good sleep quality (PSQI less than or equal 5)	119	27.5
		Poor sleep quality (PSQI more than 5)	314	72.5
PSQI components	Subjective Sleep Quality	Very Good	129	29.8
		Fairly Good	213	49.2
		Fairly Bad	73	16.9
		Very Bad	18	4.2
	Sleep Latency	No Difficulties	58	13.4
		Minimal Difficulties	177	40.9
		Fair Difficulties	133	30.7
		Severe Difficulties	65	15
	Sleep duration	> 7 Hrs.	87	20.1
		6 - 7 Hrs.	97	22.4
		5 - 6 Hrs.	198	45.7
		< 5 Hrs.	51	11.8
	Habitual Sleep Efficiency	> 85 %	317	73.2
		75 - 84 %	59	13.6
		65 - 74 %	26	6
		< 65 %	31	7.2
	Sleep Disturbance	None	21	4.8
		Minimal	291	67.2
		Fair	111	25.6
		Severe	10	2.3
	Sleep Medication	Not during past month	342	79
		Less than once a week	40	9.2
		Once or twice a week	19	4.4
		Three or more times a week	32	7.4
	Daytime Dysfunction	No Difficulties	97	22.4
		Minimal Difficulties	321	74.1
		Fair Difficulties	15	3.5
		Severe Difficulties	0	0

**Table 7:** Descriptive statistics of PSQI component among good sleep quality and Poor sleep quality groups

		GPSQI score			
		Good sleep quality (n= 119) 27.48%		Poor sleep quality (n= 314) 72.51%	
		n	%	n	%
Subjective Sleep Quality	Very Good	81	62.8%	48	37.2%
	Fairly Good	37	17.4%	176	82.6%
	Fairly Bad	1	1.4%	72	98.6%
	Very Bad	0	0%	18	100%
Sleep Latency	No Difficulties	39	67.2%	19	32.8%
	Minimal Difficulties	76	42.9%	101	57.1%
	Fair Difficulties	4	3%	129	97%
	Severe Difficulties	0	0%	65	100%
Sleep duration	> 7 Hrs.	45	51.7%	42	48.3%
	6 - 7 Hrs.	36	37.1%	61	62.9%
	5 - 6 Hrs.	36	18.2%	162	81.8%
	< 5 Hrs.	2	3.9%	49	96.1%
Habitual Sleep Efficiency	> 85 %	112	35.3%	205	64.7%
	75 - 84 %	6	10.2%	53	89.8%
	65 - 74 %	1	3.8%	25	96.2%
	< 65 %	0	0%	31	100.0%
Sleep Disturbance	None	19	90.5%	2	9.5%
	Minimal	97	33.3%	194	66.7%
	Fair	3	2.7%	108	97.3%
	Severe	0	0%	10	100%
Sleep Medication	Not during the past month	112	32.7%	230	67.3%
	Less than once a week	5	12.5%	35	87.5%
	Once or twice a week	1	5.3%	18	94.7%
	Three or more times a week	1	3.1%	31	96.9%
Daytime Dysfunction	No Difficulties	54	55.7%	43	44.3%
	Minimal Difficulties	65	20.2%	256	79.8%
	Fair Difficulties	0	0%	15	100%
	Severe Difficulties	0	0%	0	0%
